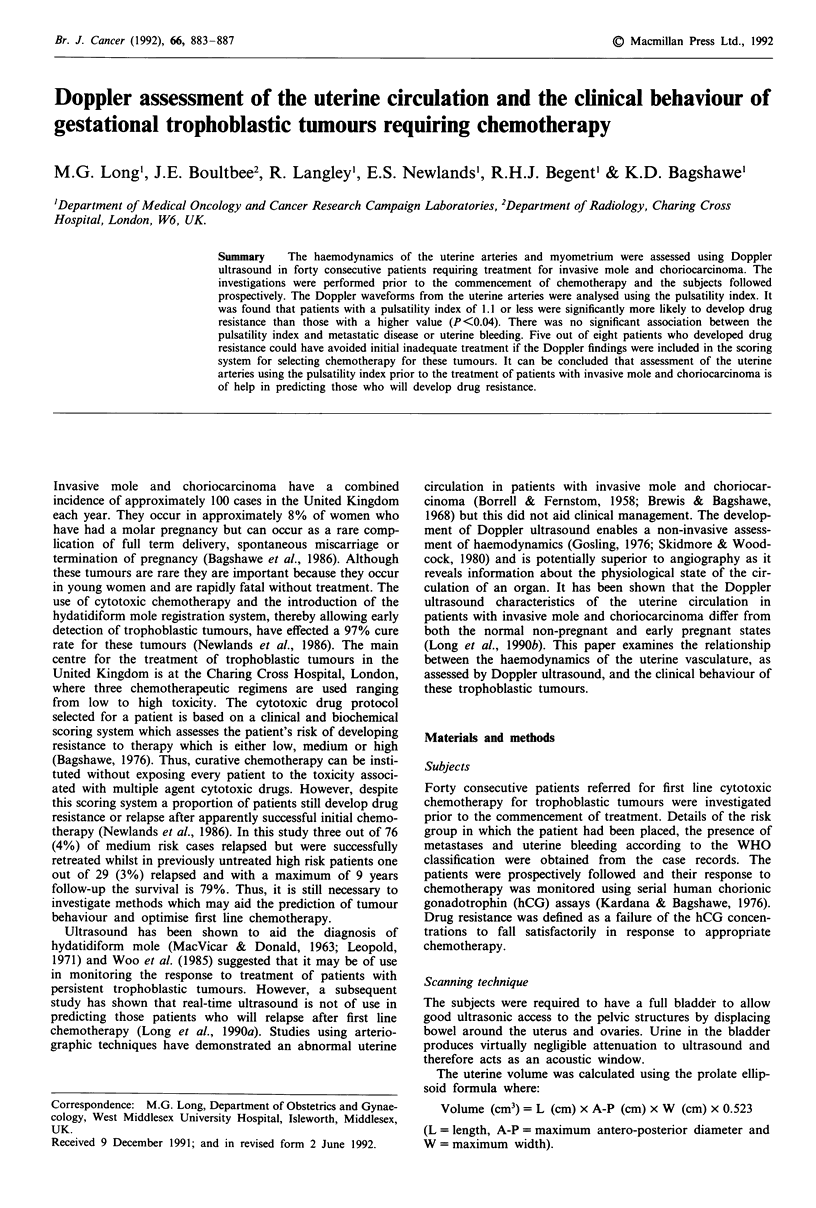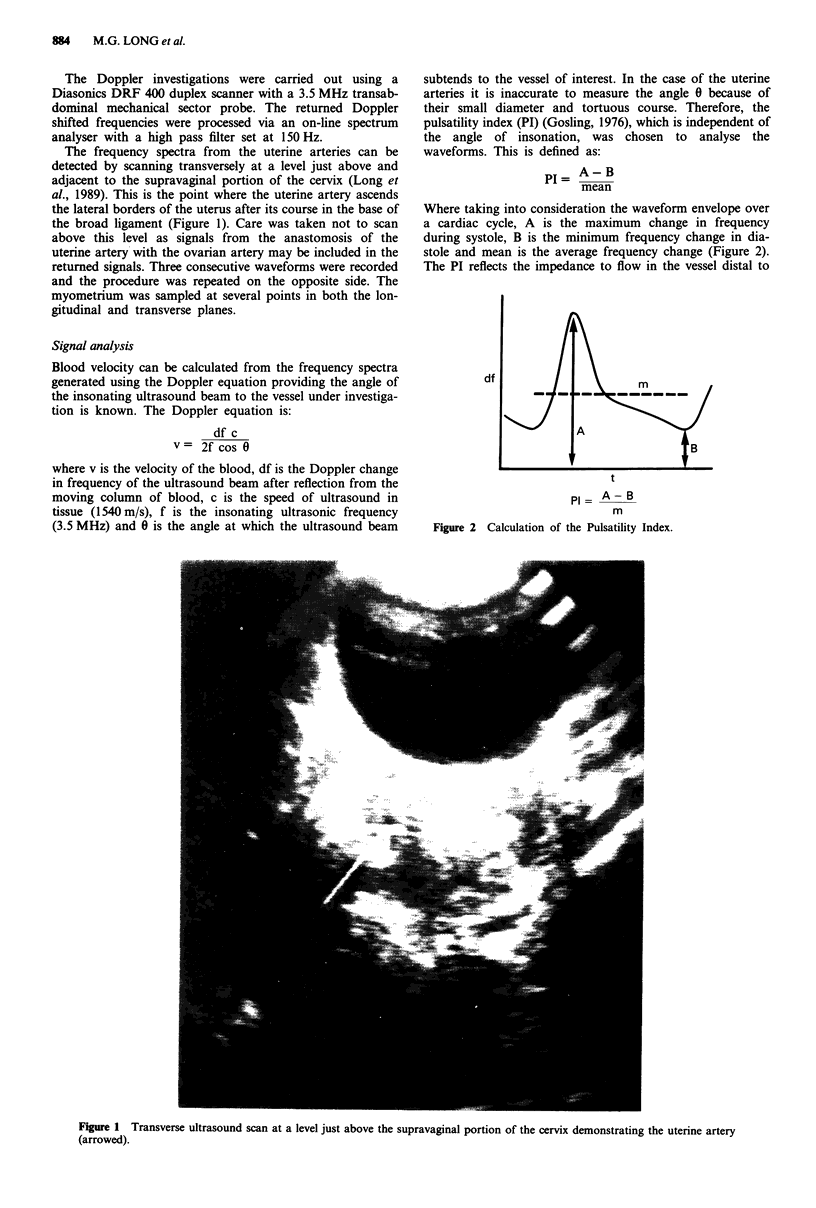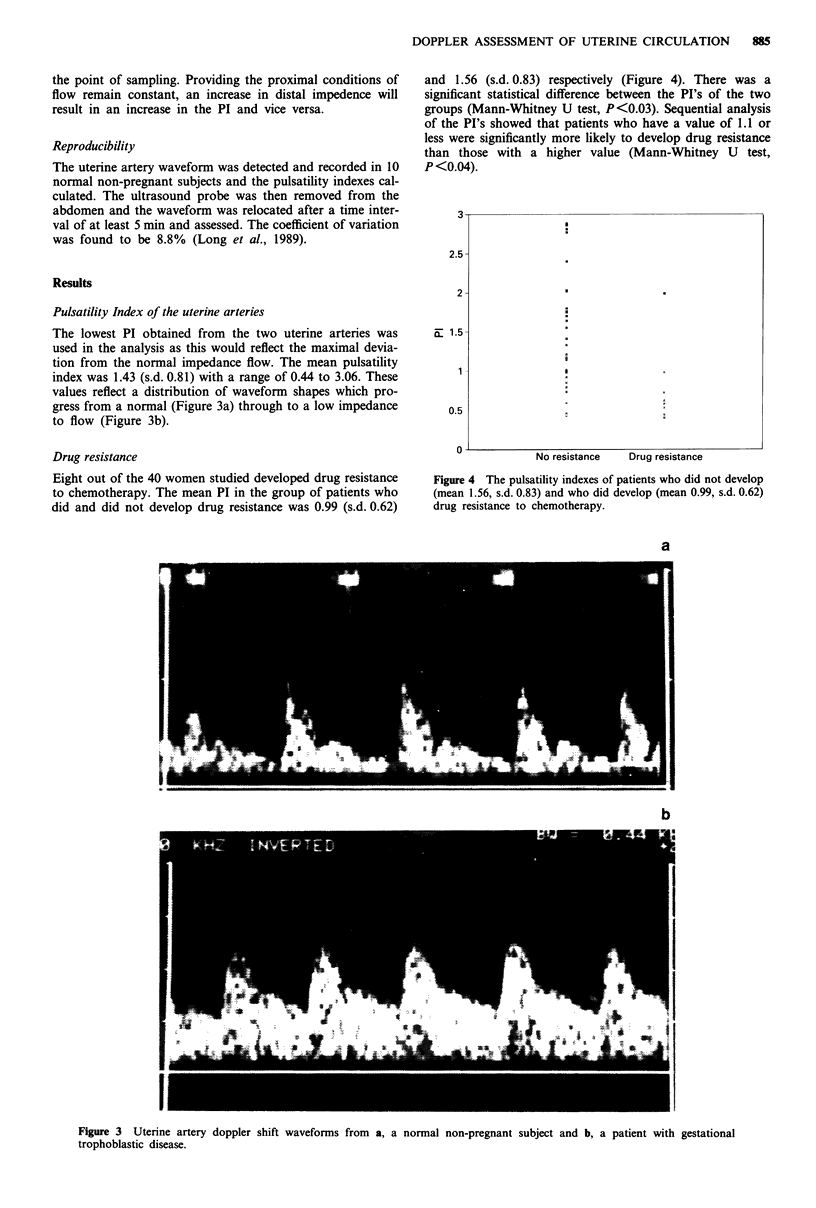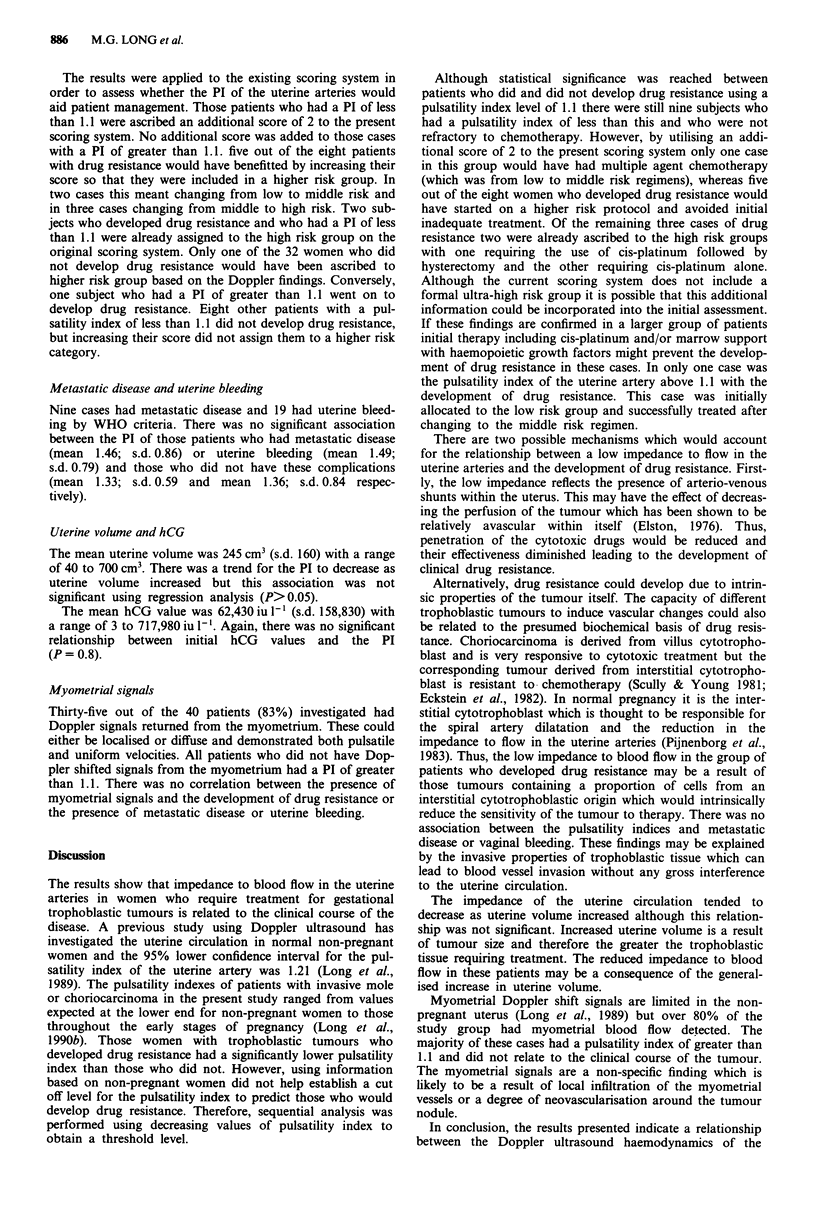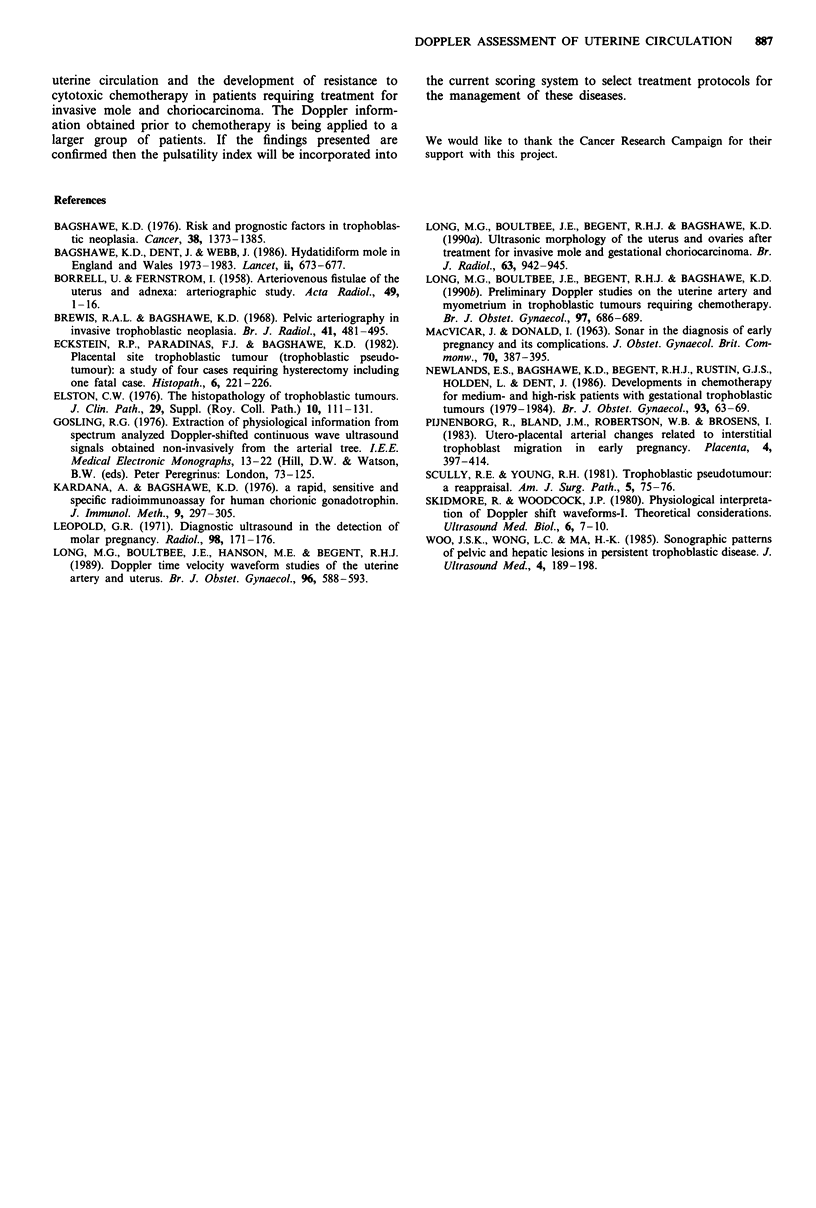# Doppler assessment of the uterine circulation and the clinical behaviour of gestational trophoblastic tumours requiring chemotherapy.

**DOI:** 10.1038/bjc.1992.379

**Published:** 1992-11

**Authors:** M. G. Long, J. E. Boultbee, R. Langley, E. S. Newlands, R. H. Begent, K. D. Bagshawe

**Affiliations:** Department of Medical Oncology, Charing Cross Hospital, London, UK.

## Abstract

**Images:**


 © Macmillan Press Ltd., 1992

### Doppler assessment of the uterine circulation and the clinical behaviour of gestational trophoblastic tumours requiring chemotherapy

M.G. Long[1], J.E. Boultbee[2], R. Langley[1], E.S. Newlands[1], R.H.J. Begent[1] & K.D. Bagshawe[1]

[1]*Department of Medical Oncology and Cancer Research Campaign Laboratories,* [2]*Department of Radiology, Charing Cross Hospital, London, W6, UK.*

**Summary** The haemodynamics of the uterine arteries and myometrium were assessed using Doppler ultrasound in forty consecutive patients requiring treatment for invasive mole and choriocarcinoma. The investigations were performed prior to the commencement of chemotherapy and the subjects followed prospectively. The Doppler waveforms from the uterine arteries were analysed using the pulsatility index. It was found that patients with a pulsatility index of 1.1 or less were significantly more likely to develop drug resistance than those with a higher value ($P<0.04$). There was no significant association between the pulsatility index and metastatic disease or uterine bleeding. Five out of eight patients who developed drug resistance could have avoided initial inadequate treatment if the Doppler findings were included in the scoring system for selecting chemotherapy for these tumours. It can be concluded that assessment of the uterine arteries using the pulsatility index prior to the treatment of patients with invasive mole and choriocarcinoma is of help in predicting those who will develop drug resistance.

Invasive mole and choriocarcinoma have a combined incidence of approximately 100 cases in the United Kingdom each year. They occur in approximately 8% of women who have had a molar pregnancy but can occur as a rare complication of full term delivery, spontaneous miscarriage or termination of pregnancy (Bagshawe *et al.*, 1986). Although these tumours are rare they are important because they occur in young women and are rapidly fatal without treatment. The use of cytotoxic chemotherapy and the introduction of the hydatidiform mole registration system, thereby allowing early detection of trophoblastic tumours, have effected a 97% cure rate for these tumours (Newlands *et al.*, 1986). The main centre for the treatment of trophoblastic tumours in the United Kingdom is at the Charing Cross Hospital, London, where three chemotherapeutic regimens are used ranging from low to high toxicity. The cytotoxic drug protocol selected for a patient is based on a clinical and biochemical scoring system which assesses the patient's risk of developing resistance to therapy which is either low, medium or high (Bagshawe, 1976). Thus, curative chemotherapy can be instituted without exposing every patient to the toxicity associated with multiple agent cytotoxic drugs. However, despite this scoring system a proportion of patients still develop drug resistance or relapse after apparently successful initial chemotherapy (Newlands *et al.*, 1986). In this study three out of 76 (4%) of medium risk cases relapsed but were successfully retreated whilst in previously untreated high risk patients one out of 29 (3%) relapsed and with a maximum of 9 years follow-up the survival is 79%. Thus, it is still necessary to investigate methods which may aid the prediction of tumour behaviour and optimise first line chemotherapy.

Ultrasound has been shown to aid the diagnosis of hydatidiform mole (MacVicar & Donald, 1963; Leopold, 1971) and Woo *et al.* (1985) suggested that it may be of use in monitoring the response to treatment of patients with persistent trophoblastic tumours. However, a subsequent study has shown that real-time ultrasound is not of use in predicting those patients who will relapse after first line chemotherapy (Long *et al.*, 1990*a*). Studies using arteriographic techniques have demonstrated an abnormal uterine circulation in patients with invasive mole and choriocarcinoma (Borrell & Fernstom, 1958; Brewis & Bagshawe, 1968) but this did not aid clinical management. The development of Doppler ultrasound enables a non-invasive assessment of haemodynamics (Gosling, 1976; Skidmore & Woodcock, 1980) and is potentially superior to angiography as it reveals information about the physiological state of the circulation of an organ. It has been shown that the Doppler ultrasound characteristics of the uterine circulation in patients with invasive mole and choriocarcinoma differ from both the normal non-pregnant and early pregnant states (Long *et al.*, 1990*b*). This paper examines the relationship between the haemodynamics of the uterine vasculature, as assessed by Doppler ultrasound, and the clinical behaviour of these trophoblastic tumours.

### Materials and methods

### *Subjects*

Forty consecutive patients referred for first line cytotoxic chemotherapy for trophoblastic tumours were investigated prior to the commencement of treatment. Details of the risk group in which the patient had been placed, the presence of metastases and uterine bleeding according to the WHO classification were obtained from the case records. The patients were prospectively followed and their response to chemotherapy was monitored using serial human chorionic gonadotrophin (hCG) assays (Kardana & Bagshawe, 1976). Drug resistance was defined as a failure of the hCG concentrations to fall satisfactorily in response to appropriate chemotherapy.

### *Scanning technique*

The subjects were required to have a full bladder to allow good ultrasonic access to the pelvic structures by displacing bowel around the uterus and ovaries. Urine in the bladder produces virtually negligible attenuation to ultrasound and therefore acts as an acoustic window.

The uterine volume was calculated using the prolate ellipsoid formula where:

$$\text{Volume (cm}^3) = \text{L (cm)} \times \text{A-P (cm)} \times \text{W (cm)} \times 0.523$$

(L = length, A-P = maximum antero-posterior diameter and W = maximum width).

Correspondence: M.G. Long, Department of Obstetrics and Gynaecology, West Middlesex University Hospital, Isleworth, Middlesex, UK.
Received 9 December 1991; and in revised form 2 June 1992.

The Doppler investigations were carried out using a Diasonics DRF 400 duplex scanner with a 3.5 MHz transabdominal mechanical sector probe. The returned Doppler shifted frequencies were processed via an on-line spectrum analyser with a high pass filter set at 150 Hz.

The frequency spectra from the uterine arteries can be detected by scanning transversely at a level just above and adjacent to the supravaginal portion of the cervix (Long *et al.*, 1989). This is the point where the uterine artery ascends the lateral borders of the uterus after its course in the base of the broad ligament (Figure 1). Care was taken not to scan above this level as signals from the anastomosis of the uterine artery with the ovarian artery may be included in the returned signals. Three consecutive waveforms were recorded and the procedure was repeated on the opposite side. The myometrium was sampled at several points in both the longitudinal and transverse planes.

*Signal analysis*

Blood velocity can be calculated from the frequency spectra generated using the Doppler equation providing the angle of the insonating ultrasound beam to the vessel under investigation is known. The Doppler equation is:

$$v = \frac{df\ c}{2f \cos \theta}$$

where v is the velocity of the blood, df is the Doppler change in frequency of the ultrasound beam after reflection from the moving column of blood, c is the speed of ultrasound in tissue (1540 m/s), f is the insonating ultrasonic frequency (3.5 MHz) and $\theta$ is the angle at which the ultrasound beam subtends to the vessel of interest. In the case of the uterine arteries it is inaccurate to measure the angle $\theta$ because of their small diameter and tortuous course. Therefore, the pulsatility index (PI) (Gosling, 1976), which is independent of the angle of insonation, was chosen to analyse the waveforms. This is defined as:

$$PI = \frac{A - B}{mean}$$

Where taking into consideration the waveform envelope over a cardiac cycle, A is the maximum change in frequency during systole, B is the minimum frequency change in diastole and mean is the average frequency change (Figure 2). The PI reflects the impedance to flow in the vessel distal to

**Figure 2** Calculation of the Pulsatility Index.

**Figure 1** Transverse ultrasound scan at a level just above the supravaginal portion of the cervix demonstrating the uterine artery (arrowed).

the point of sampling. Providing the proximal conditions of flow remain constant, an increase in distal impedence will result in an increase in the PI and vice versa.

### *Reproducibility*

The uterine artery waveform was detected and recorded in 10 normal non-pregnant subjects and the pulsatility indexes calculated. The ultrasound probe was then removed from the abdomen and the waveform was relocated after a time interval of at least 5 min and assessed. The coefficient of variation was found to be 8.8% (Long *et al.*, 1989).

### Results

### *Pulsatility Index of the uterine arteries*

The lowest PI obtained from the two uterine arteries was used in the analysis as this would reflect the maximal deviation from the normal impedance flow. The mean pulsatility index was 1.43 (s.d. 0.81) with a range of 0.44 to 3.06. These values reflect a distribution of waveform shapes which progress from a normal (Figure 3a) through to a low impedance to flow (Figure 3b).

### *Drug resistance*

Eight out of the 40 women studied developed drug resistance to chemotherapy. The mean PI in the group of patients who did and did not develop drug resistance was 0.99 (s.d. 0.62) and 1.56 (s.d. 0.83) respectively (Figure 4). There was a significant statistical difference between the PI's of the two groups (Mann-Whitney U test, $P<0.03$). Sequential analysis of the PI's showed that patients who have a value of 1.1 or less were significantly more likely to develop drug resistance than those with a higher value (Mann-Whitney U test, $P<0.04$).

**Figure 4** The pulsatility indexes of patients who did not develop (mean 1.56, s.d. 0.83) and who did develop (mean 0.99, s.d. 0.62) drug resistance to chemotherapy.

**Figure 3** Uterine artery doppler shift waveforms from **a**, a normal non-pregnant subject and **b**, a patient with gestational trophoblastic disease.

The results were applied to the existing scoring system in order to assess whether the PI of the uterine arteries would aid patient management. Those patients who had a PI of less than 1.1 were ascribed an additional score of 2 to the present scoring system. No additional score was added to those cases with a PI of greater than 1.1. five out of the eight patients with drug resistance would have benefitted by increasing their score so that they were included in a higher risk group. In two cases this meant changing from low to middle risk and in three cases changing from middle to high risk. Two subjects who developed drug resistance and who had a PI of less than 1.1 were already assigned to the high risk group on the original scoring system. Only one of the 32 women who did not develop drug resistance would have been ascribed to higher risk group based on the Doppler findings. Conversely, one subject who had a PI of greater than 1.1 went on to develop drug resistance. Eight other patients with a pulsatility index of less than 1.1 did not develop drug resistance, but increasing their score did not assign them to a higher risk category.

*Metastatic disease and uterine bleeding*

Nine cases had metastatic disease and 19 had uterine bleeding by WHO criteria. There was no significant association between the PI of those patients who had metastatic disease (mean 1.46; s.d. 0.86) or uterine bleeding (mean 1.49; s.d. 0.79) and those who did not have these complications (mean 1.33; s.d. 0.59 and mean 1.36; s.d. 0.84 respectively).

*Uterine volume and hCG*

The mean uterine volume was 245 $cm^3$ (s.d. 160) with a range of 40 to 700 $cm^3$. There was a trend for the PI to decrease as uterine volume increased but this association was not significant using regression analysis ($P > 0.05$).

The mean hCG value was 62,430 iu $l^{-1}$ (s.d. 158,830) with a range of 3 to 717,980 iu $l^{-1}$. Again, there was no significant relationship between initial hCG values and the PI ($P = 0.8$).

*Myometrial signals*

Thirty-five out of the 40 patients (83%) investigated had Doppler signals returned from the myometrium. These could either be localised or diffuse and demonstrated both pulsatile and uniform velocities. All patients who did not have Doppler shifted signals from the myometrium had a PI of greater than 1.1. There was no correlation between the presence of myometrial signals and the development of drug resistance or the presence of metastatic disease or uterine bleeding.

### Discussion

The results show that impedance to blood flow in the uterine arteries in women who require treatment for gestational trophoblastic tumours is related to the clinical course of the disease. A previous study using Doppler ultrasound has investigated the uterine circulation in normal non-pregnant women and the 95% lower confidence interval for the pulsatility index of the uterine artery was 1.21 (Long *et al.*, 1989). The pulsatility indexes of patients with invasive mole or choriocarcinoma in the present study ranged from values expected at the lower end for non-pregnant women to those throughout the early stages of pregnancy (Long *et al.*, 1990*b*). Those women with trophoblastic tumours who developed drug resistance had a significantly lower pulsatility index than those who did not. However, using information based on non-pregnant women did not help establish a cut off level for the pulsatility index to predict those who would develop drug resistance. Therefore, sequential analysis was performed using decreasing values of pulsatility index to obtain a threshold level.

Although statistical significance was reached between patients who did and did not develop drug resistance using a pulsatility index level of 1.1 there were still nine subjects who had a pulsatility index of less than this and who were not refractory to chemotherapy. However, by utilising an additional score of 2 to the present scoring system only one case in this group would have had multiple agent chemotherapy (which was from low to middle risk regimens), whereas five out of the eight women who developed drug resistance would have started on a higher risk protocol and avoided initial inadequate treatment. Of the remaining three cases of drug resistance two were already ascribed to the high risk groups with one requiring the use of cis-platinum followed by hysterectomy and the other requiring cis-platinum alone. Although the current scoring system does not include a formal ultra-high risk group it is possible that this additional information could be incorporated into the initial assessment. If these findings are confirmed in a larger group of patients initial therapy including cis-platinum and/or marrow support with haemopoietic growth factors might prevent the development of drug resistance in these cases. In only one case was the pulsatility index of the uterine artery above 1.1 with the development of drug resistance. This case was initially allocated to the low risk group and successfully treated after changing to the middle risk regimen.

There are two possible mechanisms which would account for the relationship between a low impedance to flow in the uterine arteries and the development of drug resistance. Firstly, the low impedance reflects the presence of arterio-venous shunts within the uterus. This may have the effect of decreasing the perfusion of the tumour which has been shown to be relatively avascular within itself (Elston, 1976). Thus, penetration of the cytotoxic drugs would be reduced and their effectiveness diminished leading to the development of clinical drug resistance.

Alternatively, drug resistance could develop due to intrinsic properties of the tumour itself. The capacity of different trophoblastic tumours to induce vascular changes could also be related to the presumed biochemical basis of drug resistance. Choriocarcinoma is derived from villus cytotrophoblast and is very responsive to cytotoxic treatment but the corresponding tumour derived from interstitial cytotrophoblast is resistant to chemotherapy (Scully & Young 1981; Eckstein *et al.*, 1982). In normal pregnancy it is the interstitial cytotrophoblast which is thought to be responsible for the spiral artery dilatation and the reduction in the impedance to flow in the uterine arteries (Pijnenborg *et al.*, 1983). Thus, the low impedance to blood flow in the group of patients who developed drug resistance may be a result of those tumours containing a proportion of cells from an interstitial cytotrophoblastic origin which would intrinsically reduce the sensitivity of the tumour to therapy. There was no association between the pulsatility indices and metastatic disease or vaginal bleeding. These findings may be explained by the invasive properties of trophoblastic tissue which can lead to blood vessel invasion without any gross interference to the uterine circulation.

The impedance of the uterine circulation tended to decrease as uterine volume increased although this relationship was not significant. Increased uterine volume is a result of tumour size and therefore the greater the trophoblastic tissue requiring treatment. The reduced impedance to blood flow in these patients may be a consequence of the generalised increase in uterine volume.

Myometrial Doppler shift signals are limited in the non-pregnant uterus (Long *et al.*, 1989) but over 80% of the study group had myometrial blood flow detected. The majority of these cases had a pulsatility index of greater than 1.1 and did not relate to the clinical course of the tumour. The myometrial signals are a non-specific finding which is likely to be a result of local infiltration of the myometrial vessels or a degree of neovascularisation around the tumour nodule.

In conclusion, the results presented indicate a relationship between the Doppler ultrasound haemodynamics of the uterine circulation and the development of resistance to cytotoxic chemotherapy in patients requiring treatment for invasive mole and choriocarcinoma. The Doppler information obtained prior to chemotherapy is being applied to a larger group of patients. If the findings presented are confirmed then the pulsatility index will be incorporated into the current scoring system to select treatment protocols for the management of these diseases.

We would like to thank the Cancer Research Campaign for their support with this project.

### References

BAGSHAWE, K.D. (1976). Risk and prognostic factors in trophoblastic neoplasia. *Cancer*, **38**, 1373–1385.

BAGSHAWE, K.D., DENT, J. & WEBB, J. (1986). Hydatidiform mole in England and Wales 1973–1983. *Lancet*, **ii**, 673–677.

BORRELL, U. & FERNSTROM, I. (1958). Arteriovenous fistulae of the uterus and adnexa: arteriographic study. *Acta Radiol.*, **49**, 1–16.

BREWIS, R.A.L. & BAGSHAWE, K.D. (1968). Pelvic arteriography in invasive trophoblastic neoplasia. *Br. J. Radiol.*, **41**, 481–495.

ECKSTEIN, R.P., PARADINAS, F.J. & BAGSHAWE, K.D. (1982). Placental site trophoblastic tumour (trophoblastic pseudotumour): a study of four cases requiring hysterectomy including one fatal case. *Histopath.*, **6**, 221–226.

ELSTON, C.W. (1976). The histopathology of trophoblastic tumours. *J. Clin. Path.*, **29**, Suppl. (Roy. Coll. Path.) **10**, 111–131.

GOSLING, R.G. (1976). Extraction of physiological information from spectrum analyzed Doppler-shifted continuous wave ultrasound signals obtained non-invasively from the arterial tree. *I.E.E. Medical Electronic Monographs*, 13–22 (Hill, D.W. & Watson, B.W. (eds). Peter Peregrinus: London, 73–125.

KARDANA, A. & BAGSHAWE, K.D. (1976). a rapid, sensitive and specific radioimmunoassay for human chorionic gonadotrophin. *J. Immunol. Meth.*, **9**, 297–305.

LEOPOLD, G.R. (1971). Diagnostic ultrasound in the detection of molar pregnancy. *Radiol.*, **98**, 171–176.

LONG, M.G., BOULTBEE, J.E., HANSON, M.E. & BEGENT, R.H.J. (1989). Doppler time velocity waveform studies of the uterine artery and uterus. *Br. J. Obstet. Gynaecol.*, **96**, 588–593.

LONG, M.G., BOULTBEE, J.E., BEGENT, R.H.J. & BAGSHAWE, K.D. (1990*a*). Ultrasonic morphology of the uterus and ovaries after treatment for invasive mole and gestational choriocarcinoma. *Br. J. Radiol.*, **63**, 942–945.

LONG, M.G., BOULTBEE, J.E., BEGENT, R.H.J. & BAGSHAWE, K.D. (1990*b*). Preliminary Doppler studies on the uterine artery and myometrium in trophoblastic tumours requiring chemotherapy. *Br. J. Obstet. Gynaecol.*, **97**, 686–689.

MACVICAR, J. & DONALD, I. (1963). Sonar in the diagnosis of early pregnancy and its complications. *J. Obstet. Gynaecol. Brit. Commonw.*, **70**, 387–395.

NEWLANDS, E.S., BAGSHAWE, K.D., BEGENT, R.H.J., RUSTIN, G.J.S., HOLDEN, L. & DENT, J. (1986). Developments in chemotherapy for medium- and high-risk patients with gestational trophoblastic tumours (1979–1984). *Br. J. Obstet. Gynaecol.*, **93**, 63–69.

PIJNENBORG, R., BLAND, J.M., ROBERTSON, W.B. & BROSENS, I. (1983). Utero-placental arterial changes related to interstitial trophoblast migration in early pregnancy. *Placenta*, **4**, 397–414.

SCULLY, R.E. & YOUNG, R.H. (1981). Trophoblastic pseudotumour: a reappraisal. *Am. J. Surg. Path.*, **5**, 75–76.

SKIDMORE, R. & WOODCOCK, J.P. (1980). Physiological interpretation of Doppler shift waveforms-I. Theoretical considerations. *Ultrasound Med. Biol.*, **6**, 7–10.

WOO, J.S.K., WONG, L.C. & MA, H.-K. (1985). Sonographic patterns of pelvic and hepatic lesions in persistent trophoblastic disease. *J. Ultrasound Med.*, **4**, 189–198.